# What’s the “Take Home” from Research on Dementia Trends?

**DOI:** 10.1371/journal.pmed.1002236

**Published:** 2017-03-07

**Authors:** Eric B. Larson, Kenneth M. Langa

**Affiliations:** 1 Group Health Research Institute, Seattle, Washington, United States of America; 2 Department of Medicine, Institute for Social Research, Veterans Affairs Center for Clinical Management Research, Institute for Healthcare Policy and Innovation, University of Michigan, Ann Arbor, Michigan, United States of America

## Abstract

Eric Larson and Kenneth Langa discuss whether the risk of dementia is increasing or decreasing over time.

In 2005, a study based on data from 17 years of United States National Long-Term Care Surveys reported a 42% decline in prevalence of severe cognitive impairment from 1982 to 1999 [1]. We subsequently summarized the results of this and four other studies reporting declines in prevalence or incidence of dementia in the US, England, Stockholm, and Rotterdam [2].

In *PLOS Medicine*, Emma van Bussel and colleagues report a large study on trends in age-specific dementia incidence rates from 1992 to 2014 in the Netherlands, based on primary care coding records from general practice registration networks [3]. The analysis is based on data for a large fraction of the Dutch population, and the overall conclusion drawn by the investigators was that no decline in incidence of dementia was observed. If anything, the authors note, a slight increase in the highest age groups made the greatest contribution to a small overall increase in dementia incidence (an estimated 2.1% [95% CI 0.5%–3.8%] annual increase in incidence).

The real take-home message of this important paper is that Dutch general practitioners and their communities are seeing increasing numbers of older people diagnosed with dementia, adding to the burden of work for primary care practices in an aging society. But how do we explain that other studies of European and North American communities do not see the trends reported in the Dutch study?

## Is Risk of Dementia Increasing or Decreasing?

One likely explanation is changes in medical record coding practices during the time period of van Bussel and colleagues’ study, probably driven by increased recognition of dementia by patients, families, and health care providers. In the 1980s, what is now called an “epidemic of dementia” was a “silent epidemic” [4], because the condition was often unrecognized, especially in patients over 80 years of age. While detection is still a challenge for clinicians and families, diagnoses by practitioners have clearly increased over time. The authors of the *PLOS Medicine* study wisely note that they cannot distinguish between true stabilization of incidence rates or “a balance between increased detection and a true reduction.” They correctly emphasize that we should anticipate and prepare for a future increase in dementia occurrence in countries with aging populations.

Even with an expected increase in dementia cases, we can anticipate continued variability in reported incidence and prevalence rates. Since the study by Manton and colleagues [1], a systematic review authored by Prince and coworkers has identified nine studies on dementia prevalence, eight on incidence, and four on mortality [5]. The studies reported both stable and increased rates, along with “some moderately consistent evidence to suggest that the incidence of dementia may be declining in high-income countries.” Thus, overall trends in prevalence were inconsistent across studies.

We should not be perturbed by these inconsistencies, however, since incidence and prevalence are affected by complex and often opposing factors such as diagnostic practices, population aging, socioeconomic changes that can influence general health, and changing survival time with dementia. Regardless of these challenges, it is important to know about trends in dementia prevalence and incidence over time and across countries. Why? Because for dementia and its principal causes in late life, which are Alzheimer disease and vascular brain diseases, if age is the sole factor driving increasing rates, we have little hope for prevention. However, the intriguing feature of dementia is the factors that appear to be associated with the changing rates.

Prince and colleagues suggest that increasing dementia prevalence in East Asia [5], particularly in China, is consistent with worsening cardiovascular risk profiles, with rising rates of smoking, obesity, and metabolic disease. A more recently published study not included in Prince and colleagues’ review is our analysis of data covering 2000–2012 from the Health and Retirement Study (HRS), an ongoing population-based longitudinal survey of a nationally representative sample of adults aged 51 years and older [7]. An earlier paper reported that HRS showed a significant decline between 1993 and 2002 in the prevalence of cognitive impairment among adults 70 years of age or older living in the community [6]. Our follow-up study of the HRS sample found that dementia prevalence among those 65 years of age or older decreased from 11.6% in 2000 to 8.8% in 2012. We noted that an “increase in educational attainment was associated with some of the decline in dementia prevalence, but the full set of social, behavioral, and medical factors contributing to the decline is still uncertain” [7].

## Controlling the Dementia Epidemic

Within these studies on the incidence and prevalence of dementia are hints at possible strategies to control the disease and its effects. Clearly, population aging plays the largest role in determining disease rates. We must plan for increasing numbers of predominantly older people with dementia in the decades to come, including addressing the growing need for long-term care in the context of a significant decline in the availability of family caregivers. Pressing clinical research issues include developing programs to foster activated, confident (typically family) caregivers, models for effective chronic care management, and avoiding unnecessary hospitalizations and visits to emergency rooms. New models for financing and delivering long-term care, either in community or in institutional settings, will be extremely important in the decades ahead. That said, substantial evidence supports several factors as protective against the risk of late-life dementia: advances in general education levels, especially in early life; socioeconomic well-being; and, most importantly for medical care, better control of cardiovascular risk factors, especially in midlife.

Most interesting to us is the likelihood that controlling vascular risk at the population level reduces dementia risk. International and local policies can contribute to this control if socioeconomic development results in reducing smoking and obesity and controlling diabetes and midlife hypertension. These are all important issues for further research on how to rein in the dementia epidemic.

Avoiding dementia is a strong motivator because of the wide-ranging burden of the disease on patients and families. As physicians, we find that the prospect of reducing dementia risk can be a powerful motivator for lifestyle changes as well as promoting adherence to treatment in people with diabetes, hypertension, and other sources of increased cardiovascular risk. Public health efforts to reduce these risks could have the welcome additional consequence of improving overall brain health. Dementia prevention should drive public health campaigns around beneficial lifestyle practices such as healthy eating and habitual exercise. In addition, this powerful message should be used to promote policies to provide equitable access to health care, education, and safe neighborhoods.

## Abbreviations

HRS: Health and Retirement Study
